# Supplementary material for: Dynamic decorrelation as a unifying principle for explaining a broad range of brightness phenomena
Source: PLoS Comput Biol. 2021 Apr 26;17(4):e1007907. doi: 10.1371/journal.pcbi.1007907 (PMC8102013; doi:10.1371/journal.pcbi.1007907)
Supplement: S1 Text — A. Gabor filters. In this section are described the parameter values and a mathematical description of unbalancing the ON/OFF subregions for the filters used in the Contrast-Luminance channel and the Contrast-only channel. B. Energy Map. In this section are included the mathematical details corresponding to the local energy map. C. Dynamic filtering with zero-phase whitening (ZCA). In this section are described the mathematichal details to perform dynamic filtering of our model. D. Solving Eq 5. In this section, a solution for Eq 5 is derived, which is used to estimate the output (brightness map) of our model. (PDF) [file pcbi.1007907.s001.pdf]

Supplementary Material to ”Dynamic Decorrelation as a unifying principle for explaining a broad range of brightness phenomena”

Alejandro Lerer[1], Hans Supèr[1,2,3,4], Matthias S.Keil[1,2]*,

1 Departament de Cognició, Desenvolupament i Psicologia de l'Educació, Faculty of Psychology, University of Barcelona, Barcelona, Spain
2 Institut de Neurociències, Universitat de Barcelona, Barcelona, Spain
3 Institut de Recerca Pediàtrica Hospital Sant Joan de Déu, Barcelona, Spain
4 Catalan Institute for Advanced Studies (ICREA), Barcelona, Spain

Supporting information

S1 Text.

A. Gabor filters. Mathematically, our set of Gabor filters is parameterized as:

$$g(x, y, \sigma_x, \sigma_y, \lambda, \rho) = e^{-\frac{1}{2}(\frac{\hat{x}^2}{\sigma_x^2} + \frac{\hat{y}^2}{\sigma_y^2})} \cos(2\pi \frac{x}{\lambda} + \rho)$$

where

$$\hat{x} = x\cos(\theta) - y\sin(\theta)$$
$$\hat{y} = x\sin(\theta) + y\cos(\theta)$$

ρ is the phase, the parameters (x, y) are the spatial coordinates; σ_x and σ_y are the standard deviations along x and y, respectively; λ is the wavelength; and θ the orientation. We used $\lambda \in \{4, 8\}$, eight orientations $\theta \in \{0, \frac{\pi}{4}, \frac{\pi}{2}, \ldots, 2\pi\}$, and Gabor filters with even and odd symmetry $\rho \in \{0, \frac{\pi}{2}\}$. The standard deviations σ_x and σ_y depend on the wavelength and bandwidth $bw = 2$ as $\sigma_x = \frac{\lambda}{\pi}\sqrt{\frac{\log(2)}{2}} \frac{(2^{bw}+1)}{2^{bw}-1}$ and $\sigma_y = \frac{\sigma_x}{0.64}$. For all the Contrast-only filters, the peak spatial-frequency adjusted to 0.25 cycles/pixel, while the Contrast-Luminance filters, the peak spatial-frequency adjusted to 0.125 cycles/pixel. Thus, the spatial frequency ratio between Contrast-only and Contrast-Luminance filters was 1:2.

The ON and OFF regions of a Gabor filter g are defined as positive and negative subfields, respectively:

$$\begin{aligned} g_{ON}(x, y) &= \begin{cases} g(x, y) & if \;\; g(x, y) \geq 0 \\ 0 & \text{otherwise} \end{cases} \\ g_{OFF}(x, y) &= \begin{cases} g(x, y) & if \;\; g(x, y) < 0 \\ 0 & \text{otherwise} \end{cases} \end{aligned} \quad (1)$$

Then each filter g was unbalanced by independently scaling ON and OFF regions via:

$$g = \frac{1}{2}\left[\frac{g_{ON}}{\sum_{x,y}|g_{ON}(x, y)|} + (1 - \alpha)\frac{g_{OFF}}{\sum_{x,y}|g_{OFF}(x, y)|}\right] \quad (2)$$

Notice that the ON-subfield is fixed, and the parameter only scales the OFF-subfield. With $\alpha = 0$, the area of both subfields (ON and OFF) of the filter is the same (ratio 1/1). This means that the DC component of the Gabor filter is zero (i.e., no sensitivity to mean luminance). With $\alpha \neq 0$, the ratio between both areas becomes unbalanced (with ratio $1/(1-\alpha)$) and the Gabor filters are now sensitive to mean luminance. We set $\alpha = 0.1$ for the Contrast-Luminance filters, and $\alpha = 0$ for the Contrast-only filters. With $\alpha > 0$ the contribution of ON-subfield is higher than that of the OFF-subfield. This means that the sign of luminance of a homogeneous version is preserved in the DC-response (we used luminance values from -0.5 to +0.5), because there are more positive (ON) values in the unbalanced kernel. This property is necessary for reconstruction, otherwise brightness would be predicted in the opposite direction. On the other hand, when convolved with luminance patterns, the ON amplitude (positive values) of the result will be higher than the OFF amplitude (negative values), and the difference corresponds to the DC response.

B. Energy Map. Firstly, the complex cells were computed directly from the activity of the Contrast-only channel using the local energy model [1–4] defined as:

$$C_g(x,y) = R_{g;odd}(x,y)^2 + R_{g;even}(x,y)^2$$

where $R_{g,odd}$ and $R_{g,even}$ indicates the responses from a pair of Gabor filters with identical orientation and spatial frequency, but different phase ($R_{g,even}$ with $\rho = 0$, and $R_{g,odd}$ with $\rho = \pi/2$).

Finally, the local energy map E was computed as:

$$E(x,y) = \sqrt{\sum_g C_g(x,y)}$$

C. Dynamic filtering with zero-phase whitening (ZCA). Initially, we sub-sampled the energy map E to half of its original size:

$$E_2(x,y) = \frac{E(x,y) + E(x+1,y) + E(x,y+1) + E(x+1,y+1)}{4}$$

where $x, y \in \{1, 3, 5, \ldots n-1\}$ are spatial indices. Because ZCA decorrelates intensity variations at the pixel level, the two-fold reduction in spatial scale has three advantages. First, the computational cost of ZCA is reduced. Second, the sensitivity to high spatial frequencies (and thus noise) is reduced. Third, because the edges in the energy map typically span more than one pixel in width, the scale reduction retained the intensity variations between different edges, while in turn reduced intensity variation along the edges. This led to a less variable and thus improved contour map, which in turn facilitates the decorrelation between edges when applying the ZCA method.

Next, a set of 10000 patches of size 17×17 pixels was extracted randomly from the sub-sampled energy map E_2. To be computationally tractable the set was normalized (extracting the mean and dividing by standard deviation) and cast into a matrix X of dimension $17^2 \times 10000$ such that each patch is represented by a matrix column.

The zero-phase whitening (ZCA) transformation [5] consists in finding a symmetrical matrix W of dimension $17^2 \times 17^2$ such that - after applying it to X - the spatial correlations between the patches are eliminated (i.e., the covariance matrix after transformation is equal to the identity matrix). Then, the columns of W form a base in which the patches are decorrelated. Because W is symmetric, the columns of W are identical up to cyclic shift of the elements in each column. This property permits to select any column of W, center it, and reshape it to build the kernel for the dynamic filter (see further down). In order to compute W we can solve the linear equation:

$$\mathrm{cov}(WX) = I$$

where I represents the identity matrix and cov indicates the covariance matrix. Due to W being symmetrical (i.e., $W^T = W$), after doing some linear algebraic operations, the last equation can be solved by

$$W = (X^T X)^{-1/2} = \Sigma^{-1/2}$$

where $\Sigma = \mathrm{cov}(X)$ (i.e., represents the covariance matrix of X).

However, the last equation is very sensitive to high frequencies and isolated points (usually noise). This issue can be alleviated by regularization and compression of the singular value spectrum. We thus express the covariance matrix Σ by singular value decomposition $\Sigma = USV^T$ (using Matlab's svd function) and add a regularization parameter according to $\Sigma = U(S + \epsilon I)V^T$, where ϵ was set to $0.01 * \max(S)$ and I is the identity matrix, and S is the matrix with singular values along its diagonal. In addition we reduce the dimension of the singular matrix S such that only the highest 50 singular values were kept and the remaining values were set to zero. The compressed matrix is $\tilde{S}$, and we have

$$W = U(\tilde{S} + \epsilon I)^{-1/2} V^T$$

By imposing symmetry (V is substituted by U) we arrive at

$$W = U(\tilde{S} + \epsilon I)^{-1/2} U^T$$

We define the dynamic filter F as the column of W with index $\frac{17^2+1}{2}$ which corresponds to the centered column relative to the patches. The dynamic filter is scaled up (using Matlab's imresize) and reshaped (using Matlab's reshape) into a 2-dimensional matrix with size $(2 \times 17 - 1) \times (2 \times 17 - 1)$. Finally, the filter was normalized as $F(x, y) := \frac{F(x,y)}{\sum_{x,y} |F(x,y)|}$ to be computationally tractable.

D. Solving Eq 5.

The following gradient descent method served to compute the minima of the objective function $E(z)$ of Eq 5. Note that mathematically the Eq 5 is convex and therefore has a global minimum (i.e., a unique minimum). The discretized gradient descent is

$$z_{k+1} = z_k - \eta \nabla_z E(z)$$

where $\eta = 0.1$ is a learning parameter. The method terminates either when having reached a maximum number of iterations (100 in our simulations), or when an error criterion was satisfied:

$$\frac{\| z_{n+1} - z_n \|^2}{\| z_{n+1} \|^2} < 10^{-6}$$

The last formula measures the difference in reconstruction between subsequent iterations ("relative error"). In terms of convergence, we observed as our model converged (globally) to a stable solution in all considered cases.

Because the objective function involves a norm and a linear operator (convolution), we computed the gradient $\nabla_z E$, indirectly, by using the definition of the directional derivative D_v as:

$$D_vE(z) = \lim_{\epsilon \to 0} \frac{E(z+\epsilon v) - E(z)}{\epsilon}$$

$$\lim_{\epsilon \to 0} \frac{\sum_g \parallel R_g^* - g * (z+\epsilon v) \parallel^2 + \mu \parallel \nabla^2 (z+\epsilon v) \parallel^2 - \sum_g \parallel R_g^* - g * z \parallel^2 - \mu \parallel \nabla^2 z \parallel^2}{\epsilon}$$

$$= \lim_{\epsilon \to 0} \frac{\sum_g \langle R_g^* - g * (z+\epsilon v), R_g^* - g * (z+\epsilon v) \rangle + \mu \langle \nabla^2 (z+\epsilon v), \nabla^2 (z+\epsilon v) \rangle}{\epsilon}$$

$$- \frac{\sum_g \langle R_g^* - g * z, R_g^* - g * z \rangle + \mu \langle \nabla^2 z, \nabla^2 z \rangle}{\epsilon}$$

with the scalar product $\langle x, x \rangle = \parallel x \parallel^2$. After a straightforward manipulation of the scalar products and applying the limit we obtain:

$$= \sum_g -2\langle R_g^*, g * v \rangle + 2\langle g * z, g * v \rangle + 2\mu \langle \nabla^2 z, \nabla^2 v \rangle \tag{3}$$

Now, isolating the directional vector v in the scalar product

$$= \sum_g -2\langle \overline{g} * R_g^*, v \rangle + 2\langle \overline{g} * g * z, v \rangle + 2\mu \langle \overline{\nabla^2} \nabla^2 z, v \rangle$$

where filters with a bar indicate the corresponding complex adjoint. In particular, it holds:

$$\overline{\nabla^2} = \nabla^2$$

whereas taking the adjoint of Gabor filter g correspond to the same Gabor filters (as defined above in **??**) but with opposite sign of the phase parameter i.e., $\rho \in \{0, -\pi\}$. Finally using the the identity $D_vE(z) = \langle \nabla_z E_(z), v \rangle$ for the directional derivative, we obtain:

$$\nabla E_z(z) = \sum_g -2\langle \overline{g} * R_g^*, v \rangle + 2\langle \overline{g} * g * z, v \rangle + 2\mu \langle \overline{\nabla^2} \nabla^2 z, v \rangle \tag{4}$$

References

1. Morrone MC, Owens RA. Feature detection from local energy. Pattern Recognit. Lett. 6 (1987): 303-313.

2. Morrone MC, Burr DC. Feature detection in human vision: a phase-dependent energy model. Proc R Soc Lond B Biol Sci. 1988;235(1280):221-245. doi:10.1098/rspb.1988.0073

3. Adelson EH, Bergen JR. Spatiotemporal energy models for the perception of motion. J. Opt. Soc. Am. 1985; A 2, 284-299

4. Pollen DA, Ronner SF. Visual Cortical Neurons as Localized Spatial Frequency Filters. IEEE Transactions on Systems, Man, & Cybernetics. 1983; 13(5), 907–916. https://doi.org/10.1109/TSMC.1983.6313086

5. Bell AJ, Sejnowski TJ. The “independent components” of natural scenes are edge filters. Vision Res. 1997;37(23):3327-3338. doi:10.1016/s0042-6989(97)00121-1
