# Supplementary material for: Dynamic decorrelation as a unifying principle for explaining a broad range of brightness phenomena
Source: PLoS Comput Biol. 2021 Apr 26;17(4):e1007907. doi: 10.1371/journal.pcbi.1007907 (PMC8102013; doi:10.1371/journal.pcbi.1007907)
Supplement: Attachment — Submitted filename: Replies_ALLv2.pdf [file pcbi.1007907.s002.pdf]

“Dynamic decorrelation as a unifying principle for explaining a broad range of brightness phenomena” by A. Lerer, H. Super, & M. S. Keil

All of us wish to express our sincerest gratitude to the three reviewers for their time to carefully read our manuscript, and for their suggestions for improving it.

Replies to Reviewer 1

The manuscript describes a new version of an edge-integration model of brightness perception and shows that it gives a good account of a wide range of brightness/phenomena/illusions. Edge-integration, or “filling-in” models of brightness/lightness perception have a long history in visual science, beginning with the Retinex model of Land & McCann (cited) and more recently in a series of studies by Michael Rudd, none of which curiously are cited or discussed in the present article.

Our understanding is that Rudd’s model is a modification of classical retinex to incorporate distance-dependent interactions and an instruction-dependent parameterization. Unlike ours and more recent advances of retinex based on variational formulations, it is difficult to judge how Rudd’s model would perform with real-world images. This ability is, however, a distinct feature of our model. The same holds also true for many other published edge integration models, with exceptions that we already cited. We nevertheless added the following references/citations to the introduction:

Retinex at 50: color theory and spatial algorithms, a review
John J. McCann
Journal of Electronic Imaging 26(3):031204 (2017)
https://doi.org/10.1117/1.JEI.26.3.031204

Lightness computation by the human visual system
Rudd, M.
Journal of Electronic Imaging 26(3):031209 (2017)
https://doi.org/10.1117/1.JEI.26.3.031209

The proposed model variant emphasises the incorporation of predictive (or probably better termed efficient) coding, specifically filter response equalization, and the title of the manuscript suggests that this is the new unique feature of the model. However it is not clear to this reviewer in what way it is new given that many existing models of brightness perception incorporate contrast/response normalization in their model architectures.

We used ZCA for computing the dynamic filter, and we agree that predictive coding is maybe a too general description for it. As an alternative, we suggest the terms dynamic decorrelation or response equalization method, respectively. ZCA is certainly a unique feature with respect to other brightness models. We changed the corresponding occurrences in the text. However, we pointed already out in the introduction that predictive coding is a very general information processing principle, which can be implemented by many possible algorithms (e.g. Spratling, M., Brain and Cognition 112. https://doi.org/10.1016/j.bandc.2015.11.003).

We also agree with the reviewer in that the use of the term “normalization” is somewhat unfortunate (in the sense of *feature normalization*), as a corresponding mechanism should at least fulfill two criteria: *(1)* a constrained target range R *(2)* a mapping operation to R. Accordingly, a simple example is a sigmoid, or linear re-scaling $(x_i - min(x_i))/(max(x_i) - min(x_i))$, but also the membrane potential equations typically used by S. Grossberg implement an implicit response normalization. We used the term normalization to describe the effect of the denominator in Equation 4. According to the two criteria, this is therefore not a *feature normalization* mechanism, so we instead refer to it as *modulation term* in the revised version of our manuscript.

Having said that the model’s predictions of a range of brightness illusions are well-described and convincing. I was particularly impressed by the ability of the model to predict the disappearance of Hermann grid spots in a corrugated grid.

Thank you!

Whether the proposed model variant of edge-integration/filling-in will have any impact on a field that is already replete with models of brightness phenomena is hard to say, but I see no reason why the model should not be published. All I can do here is to suggest some possible ways to make the manuscript more focused and digestible.

Without having counted it exactly, it appears that multi-scale approaches are more common than FI-approaches. This may also be due to the fact that FI-models are mathematically more sophisticated than typical multi-scale filter approaches.

1. Although it is commendable that in their Introduction the authors attempt to summarise most of the existing approaches to studying brightness phenomena it is perhaps too tall an order to attempt to do this in a non-review article.

We agree. Our original intention was to present some recent modeling approaches that are related to our model. As you commented, it not possible to review each and every model due to space constraints. We focussed on computational approaches, but omitted earlier 2D (and even more 1D-models) or non-computational proposals or demonstrations (but see below).

The authors in any case omit to mention some important approaches, for example the “Intrinsic-image” approach best associated with the work of Adelson/Anderson/Ekroll/Logvinenko and others, and the “Feature model” approach best associated with Watt/Morgan/Morrone/Burr/Georgeson and others.

We included (and extended on) some of the suggestions, and re-wrote the corresponding parts of the introduction.

Intrinsic image approaches (added references)

- Motoyoshi, I., Nishida, S., Sharan, L., & Adelson, E.H. (2007). Image statistics and the perception of surface qualities. Nature 447:206-209
- Tappen, M.F., Freeman, W.T. & Adelson, E.H. (2005) Recovering Intrinsic Images from a Single Image. IEEE PAMI 27(9):1459-1472
- Schmid, A. C. & Anderson, B. L. (2017) Perceptual dimensions underlying lightness perception in homogeneous center-surround displays. Journal of Vision 17(2):6
- Anderson, B. L., Whitbread, M., & de Silva, C. (2014). Lightness, brightness, and anchoring. Journal of Vision 14:7
- Anderson, B. L., Winawer, J. (2005) Image segmentation and lightness perception. Nature 434:79-83 (2005)
- F. Faul, F. & Ekroll, V. (2011) On the filter approach to perceptual transparency. Journal of Vision 11:7

- Rother, C., Kiefel, M. Zhang, L., Schölkopf, B. & Gehler, P.V. (2011). Recovering Intrinsic Images with a Global Sparsity Prior on Reflectance (NIPS 24, 2011)

Feature model approaches (Morrone/Burr were already referenced in our previous version):

- Kingdom, F. & Moulden, B. (1992) A multi-channel approach to brightness coding. Vision Research 32(8):1565-1582 (1992)
- Watt, R.J. & Morgan, M. (1985 A theory of the primitive spatial code in human vision. Vision Research 25:1661-1674
- du Buf, J.M.H. and Fischer, S. (1995). Modeling brightness perception and syntactical image coding . Optical Engineering 34(7):1900-1911

As a suggestion it might be better to alert the reader in the Introduction to sources that provide details of existing approaches to brightness/lightness perception (e.g. Gilchrist's book "Seeing Black and White"; Kingdom's 2011 review article in Vision Research), …

We included:

- Grossberg, A. (2017) The visual world as illusion: The ones we know and the ones we don't. In Oxford Compendium of Visual Illusions (A. Shapiro and D. Todorovic, Eds.)
- Kingdom, F.A.A. (2011) Lightness, brightness and transparency: A quarter century of new ideas, captivating demonstrations and unrelenting controversy. Vision Research 51(7):652-673

We streamlined/shortened the descriptions of the already included models.

…and concentrate instead on providing the rationale for the edge-integration approach in the context of its many existing model variants and the critical empirical evidence that speaks to its validity.

We hope that the new version of the introduction is satisfactory in this respect. In the introduction, we excluded the paragraph about anchoring theory, because It is not directly related to our model. Instead, we emphasized further that edge-integration models act to suppress illumination effects, and suggested solutions to contradictions with respect to neuronal spreading and filling-in approaches, respectively. These apparently contradicting claims are often made in the context of Grating Induction and additive noise. Accordingly, we believe that FI cannot be discarded (yet) as an alternative to multi-scale filtering.

2. The main pitch is "predictive coding". This appears to be implemented in the proposed model by selective enhancement of edges via response equalization. Yet response equalization is a feature of many existing models of brightness perception. In the case of multiscale filtering models it is a critical feature of, for example, Blakeslee & McCourt's ODOG model (cited), Dakin & Bex's model of the Cornsweet Illusion (not cited: Proc. Roy. Soc. B., 2003, 270) and Kingdom's model of Mach Bands (cited).

We suppressed the term "*predictive coding*" (see our first answer). We now also avoid the term "*normalization*", because "n*ormalization*", "*contrast equalization*", "*gain control*" etc. are very general terms, which can be defined mathematically in many different ways. This means that when only comparing models according to these general terms, it is near to impossible to propose any new kind of mechanism. The danger of such generalization is also evident in that the authors of the ODOG model refer to "*normalization*" to what you call "*contrast equalization*". According to our understanding , "*contrast normalization*" and "*equalization*" are basically different concepts (although they might lead to similar results): In the ODOG model, the re-weighting across scales is essentially contrast equalization. The division by the rms level is referred to as normalization. However, in terms of neuronal computations, we think that "*modulation*" or "*gain control*" would have been a more adequate description.

Dakin & Bex admitted that their model is essentially a version of ODOG based on isotropic filters. We included it in the introduction as you recommended. They refer to their mechanism as "*normalization*", while for their model "*response equalization*" would be more adequate.
Also, Kingdom's model of Mach Bands implements normalization by "*multiplying each filter response by a constant*" (Eq.2). Therefore, Kingdom did apply a weight to filter responses.
The examples show that such an inflationary use of "*normalization*" is not helpful for comparing the mechanisms of different models.

In the case of edge-integration models contrast gain control is a feature that runs throughout the models of Rudd & colleagues (e.g. Rudd, JOV, 2010, 10(14):40; Rudd, JOV, 2013, 13(14):18; Rudd, J. Electronic Imaging, 2017, 26(3)), though whether the contrast gain adjustments in Rudd's models constitute examples of predictive coding is debatable. If the authors want to make the case that predictive coding is a novel feature of their model they need to explain in what way it is novel given existing models of brightness perception that also incorporate response/contrast equalization.

As said, predictive coding and normalization cannot represent novel features unless we compare different mechanisms at the mathematical level. For example, response equalization as used by *Dakin & Bex* could as well be considered as predictive coding, since it uses information about natural images for equalizing filter responses. The term "*predictive coding*" does no longer appear in our manuscript.

3. A critical property of the proposed model is "filling-in". There has certainly been some recent evidence that favors the filling-in approach over multi-scale filtering models, for example the study by Betz et al. (cited). It would help the reader if this evidence was flushed out with a bit more detail in the Introduction as part of the rationale for adopting an approach that is still highly controversial.

This suggestion is now addressed in the introduction, as already described above in the context of edge-integration models. Filling-in models are considered controversial because of some questionable claims that were made with Grating Induction. These claims are debunked in our the new version of the introduction.

4. The authors might want to speculate as to the function of the low-spatial-frequency filters in the cortex that play no part in their model of brightness perception, i.e. those that are lower in spatial frequency than the "contrast-luminance" channel in their model.

One of us proposed (https://doi.org/10.18725/OPARU-34) that simple cells are suited to analyze the visual input in terms of surface contours (odd simple cells with small receptive fields), texture elements (even simple cells with small receptive fields), and illumination effects (shadows and shading – simple cells of both symmetries with big receptive fields). Therefore, simple cells with a preference for lower spatial frequencies would encode smooth luminance gradients. A corresponding model could explain quantitatively many data on Mach Bands and the perception of light emission (https://doi.org/10.1162/089976606775774705, https://doi.org/10.1016/j.visres.2007.09.018). We also proposed a model which combines this idea with surface representations (https://doi.org/10.1016/j.neunet.2018.11.001).

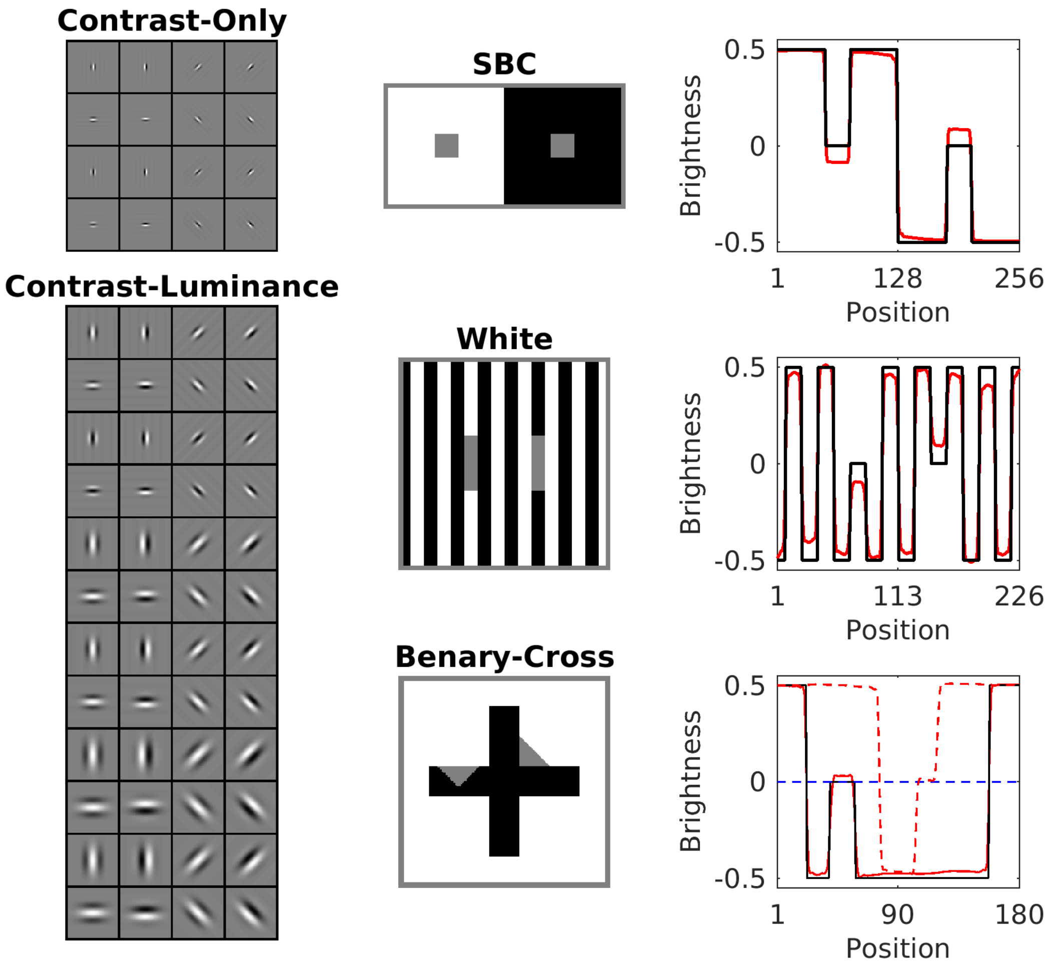

Figure R1: Left: (top part) Gabor filter set of the contrast-only channel with spatial frequency of 0.25 cycles/pixel. (Bottom part) Three sets of Gabor filters for the contrast-luminance channel with spatial frequencies 0.125 cycles/pixel, 0.0625 cycles/pixel, and 0.03125 cycles/pixel, respectively. **Middle:** SBC, White's effect and Benary Cross. **Right**: Profiles of brightness estimation computed with the three sets of contrast-luminance filters. In each plot, the three curves overlap.

To the best of our knowledge, there is no evidence for an explicit or direct (spatial) LP-pass channel (there are cells that respond to temporal changes in luminance, though). An explicit LP-channel is a crucial feature in some models nevertheless. One of us showed that in fact isotropic high-pass information is sufficient to decode natural images (https://doi.org/10.1016/j.neunet.2005.04.003). This means that from a perspective of information processing, the brain would not require low-pass sensitive cells for surface perception. LP information could, however, accelerate a filling-in process. While this might be less relevant for static displays, it may be crucial for motion perception. Now the question is how to encode LP information. One possibility is to use unbalanced retinal ganglion cells as suggested in https://doi.org/10.1016/0893-6080(96)00023-8. Our approach was to use unbalanced oriented filters in the contrast-luminance channel. We run some additional simulations with bigger filter kernels. With these we were unable to identify significant changes across the set of simulated images. Figure R1 compares the reconstruction based on three sets of contrast-luminance cells with different spatial frequencies. Since bigger

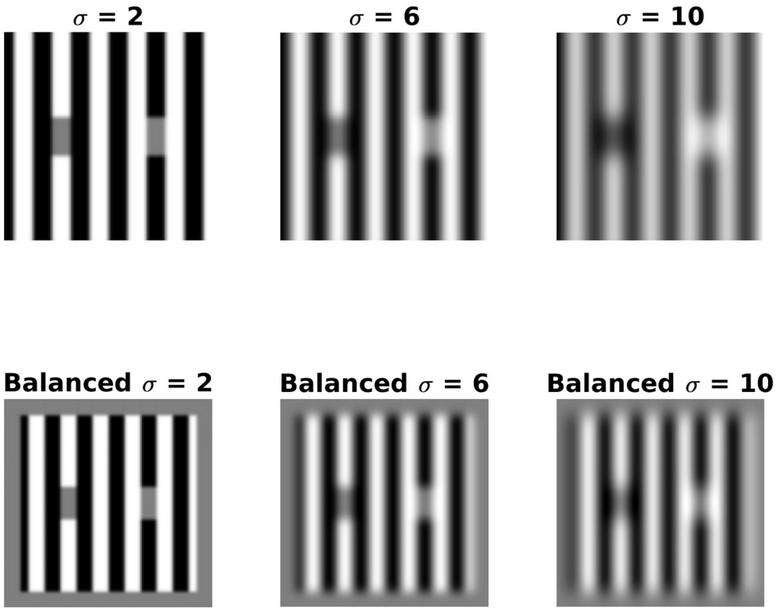

Figure R2: Top-row: White's effect lowpass-filtered with Gaussian kernels of standard deviation 2, 6, and 10. Bottom-row: Same as top, but the gray patches were corrected such that they physically have the same luminance (as with the original = unfiltered display). We think that the reviewer would agree with that there is no clear enhancement of the illusion in the bottom row.

filter kernels increase the computational cost, we adopted the smallest possible big kernel for reproducing our results.

A related issue worth commenting upon is this: if brightness perception is so dependent on the presence of edges detected by high-resolution filters, why are brightness illusions often enhanced by lowpass filtering (e.g. grating induction – see McCourt & Blakeslee, 1993, Vis. Res., 33, No. 17; White's Effect – try it yourselves!)?

A look at Figure 2 in McCourt & Blakeslee, 1993, Vis. Res., 33, No. 17 suggest that the effect is not very clear, and with only two subjects in the study, any conclusions with respect to brain mechanisms stand on rather weak feet in our opinion. Moreover, a high pass filter would still respond to blurred contours, although with reduced amplitude. This renders the conclusion of the paper that *"filters selectively sensitive to high spatial frequencies are not essential for GI"* also somewhat questionable.

It is well known that lowpass filtering reproduces assimilation effects (considering for the purpose of this answer White's effect as one of them). So we are faced with a similar "dilemma" as in McCourt & Blakeslee, 1993, Vis. Res., 33, No. 17: Applying too much filtering introduces the effect physically into the display, while applying only a small amount may not significantly alter its perceived strength. We illustrate this point with Figure R2, where we juxtapose lowpass-filtered results with the same lowpass-filtered results but with the patches having the same luminance. With the latter corrected displays, we are unable to perceive any clear enhancement.

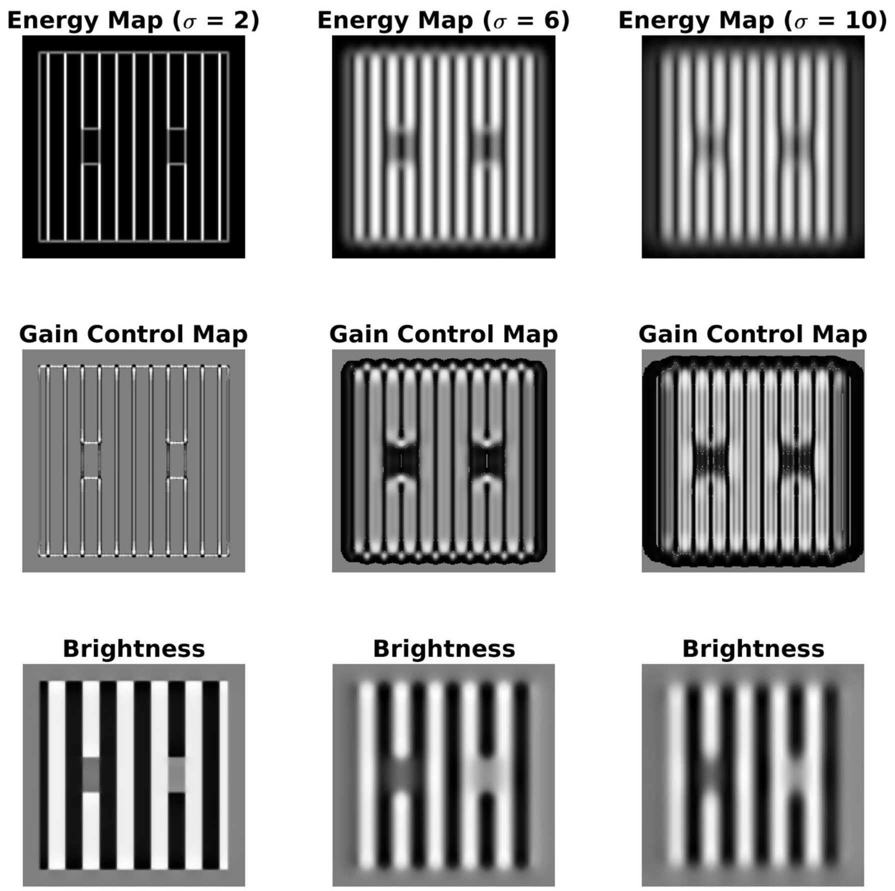

Figure R3: Top row: Energy maps for the bottom row of Figure R2. Middle row: Gain control maps. Bottom row: Brightness estimations (model output).

Our model will still work despite of high spatial frequencies being absent from the input. This is because the contrast-only channel remains sensitive to luminance variations also at lower than the highest spatial frequencies. Thus, an energy map is still estimated (Figure R3, first row). The predicted brightness of the corrected displays are shown in the last row of Figure R3. Additional simulations with uncorrected patches reproduce your mentioned brightness enhancement (Figure R4). The explanation proceeds according to Scenario 2 (Section 2.4 in the manuscript): Lowpass filtering of White's display increases the redundancy between the vertical edges of the gray patches. This leads to increased inhibition of the gain control map after dynamic filtering.

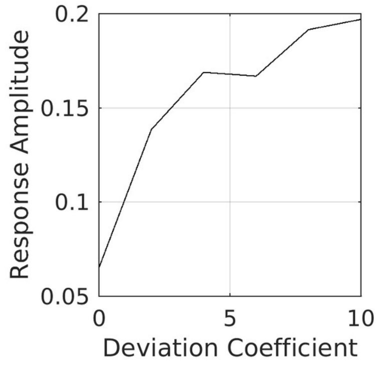

Figure R4: Positive values indicate assimilation of the patches as predicted by our model as a function of lowpass-filtering of White's display (abscissa). "Deviation Coefficient" denotes the standard deviation of the Gaussian kernel that was used for lowpass filtering.

We wish to point out that it was not our primary objective to determine which spatial frequencies are crucial for perceiving certain illusions. Our focus was rather to propose that contours play a fundamental role in predicting (at least some) brightness phenomena. Although the relation between spatial frequency content and perceived strength of brightness illusions is certainly an interesting topic to delve into, it is out of the intended scope of our study.

A brief description of the results shown here is now also included in Section 2.1.1. of our manuscript.

5. Although the “boundary contour system (BCS)” and “feature contour system (FCS)” referred to by the authors are initially presented as hypothesised model features, they rapidly morph in the text to the status of established facts. They are not facts and should only be referred to as possible mechanisms.

We made it clearer that these are merely hypothetical mechanisms. But we also added several references which support the notion that BCS/FCS is a plausible mechanisms in the visual system:

Chen, S and Glasauer, S and Mueller, HJ and Conci, M (2018) Surface filling-in and contour interpolation contribute independently to Kanizsa figure formation. Journal of Experimental Psychology. Human Perception and Performance 44(9):1399-1413

Breitmeyer, B. G., Kafalıgönül, H., Öğmen, H., Mardon, L., Todd, S., & Ziegler, R. (2006). Meta-and paracontrast reveal differences between contour-and brightness-processing mechanisms. Vision Research, 46(17), 2645-2658.

Chou P. Hung, Benjamin M. Ramsden, Li Min Chen and Anna Wang Roe (2001) Building surfaces from borders in Areas 17 and 18 of the cat. Vision Research 41(10-11):1389-1407

Lamme, V.A.F., Rodriguez-Rodriguez, V., Spekreijse, H (1999) Separate Processing Dynamics for Texture Elements, Boundaries and Surfaces in Primary Visual Cortex of the Macaque Monkey. Cerebral Cortex 9:406-413

Diane C. Rogers-Ramachandran, D.C. & Ramachandran, V.S. (1998) Psychophysical evidence for boundary and surface systems in human vision. Vision Research 38:71-77

6. Page 2 “Typically, multi-scale models adjust the response amplitudes….in a way that follows the shape of the contrast sensitivity function”. If by contrast sensitivity function you are referring to the threshold version this statement is both incorrect and a trivialisation of multi-scale models, at least for the versions mentioned in point 2 above. Best to check the details of these models before making this claim.

Thank you for pointing out this error in the manuscript. In fact, only the model of Otazu *at al.* (2008) uses a context-sensitive and adaptive version of the CSF. We corrected the corresponding text passage.

7. A key feature in the proposed model is the use of just two scales of filtering: high resolution “contrast-only” and low resolution “contrast-luminance” filters, the latter instantiated by filters that are not dc-balanced. First, what are the peak spatial-frequencies of the two types of channel expressed in cycles-per-image, and hence what is their spatial-frequency ratio? The answer to this question will help the reader understand the range of filter scales that have been omitted from the model computations, and thus be in a position to make up their own mind as to whether they consider this to be realistic.

Your statement appears to presume that neurons and our filter kernels have a near sudden cut-off in their frequency response (frequency bandwidth against zero). In signal processing, filters with sharp cut off introduce artifacts in their responses. Furthermore, the brain would need an infinite

number of filters if the frequency bandwidth of each one is near zero, yielding maximum sparsity in frequency space. Therefore, it is sound to assume that filters in the brain use a gradual roll off with respect to their preferred frequency. We also used such filters in our model. As explained above with the highpass filter (Laplacian), one gets responses to many spatial frequencies from such kernels, not only to the preferred one. The non-preferred spatial frequencies will come at lower response amplitudes (with amplitudes decreasing with increasing deviation), which could be amplified by post-processing. The already mentioned paper (https://doi.org/10.1016/j.neunet.2005.04.003) demonstrated that isotropic highpass information is sufficient to completely reconstruct natural images (i.e., also the lower spatial frequencies!).
As requested, we included the spatial frequency figures (0.25 cycles/pixel and 0.125 cycles/pixel, respectively) in Figure 2 of the manuscript, and (as mentioned above) in Section 2.1.1., as well as in Appendix A.1.

Second, in the proposed model, the spreading of neural activity in the filling-in process appears to be instantiated by the low-resolution contrast-luminance channel, yet even after multiple readings I cannot fathom what exactly is the role of the contrast-only filters in the filling-in process. Do they act as borders to gate the extent of filling-in, or what?

The spreading of neural activity in the filling-in process is instantiated by both channels (i.e., not only contrast-luminance) even though the contribution of the contrast-luminance channel is much higher because

1. it involves lower frequency filters (*https://doi.org/10.1016/j.neunet.2018.11.001*)
2. the residual luminance responses leads to a further reduction of iterations to satisfy the stop criterion (see Figure R6).

Similarly, both channels act as diffusion barriers because they both encode contrast. The contrast-only filters could be excluded without significant changes in the results. However, the contrast-only channel reduces both under-sampling and artifacts that tend to accumulate close to edges (new Figure 6C vs. 6D in the manuscript).

We have included parts of this answer in section 2.3 Stage 3: Brightness estimation filling in-process and also added Figure 6.

8. Stylistic. I have not come across the approach of using a reference number as the subject of a sentence or clause, e.g. “More recently, [28] extended the mechanism…” Is this a style recommended by PLoS? If not this needs fixing throughout. Also, the introduction of author names without prior referencing is very confusing, e.g. “Thus, Domijan modified etc.”

We added the author names to the reference numbers where applicable.

Replies to Reviewer 2

Authors present a " a hypothetical information processing strategy for visual system" (L726) which, from a raw visual input, outputs an estimate of a perceived brightness value. This model is then applied to different brightness illusions and fitted to their qualitative effects. While the paper and results are interesting, I have some points of concerns which necessitate a major revision. I am first concerned by the clarity of the presentation. For instance the introduction uses terms which may not be customary to the readership of PLoS CB but more to vision neuroscientists (eg L53 Eigengrau, L78 Benary cross). A synthetic figure which would present the different brightness illusion displays that are used in the text would be better justifying the point of the paper and simplifying the presentation of the introduction. A similar strategy applies to the rest of the manuscript (+ check syntax, eg beware of using mixed verb tenses as is the case in the author's summary).

We added Figure 1 to the introduction which shows some of the illusions to which the introduction refers to. The term “Eigengrau” does not appear anymore in the revised version, as the corresponding paragraph has been taken out. The decision of suppressing the paragraph has been taken on grounds of the comments of R1, but also that the anchoring theory is not strictly relevant for our model. The rest of the suppressed paragraph has been moved to other parts of the introduction. The introduction has been re-written in large parts, and we hope that its new version is better understandable.

My biggest concern though is scientific. How do you justify the derivation of your model? There are lots of phenomenological models like yours, and some are dealing with the same problem that you try to solve. For instance, you may check recent papers by Dario Prandi (eg https://journals.physiology.org/doi/full/10.1152/jn.00488.2019). First, there should be a justification for the choice of some parameters (eg threshold (L268) and sigmoid (L270) ...) or at least describe what would happen if you change these.

The reference https://journals.physiology.org/doi/full/10.1152/jn.00488.2019 appears not to be an adequate model for comparing it with ours: (p. 1610, and Table 1) “*The parameters appearing in the models have been chosen independently for each illusion and each model, to obtain the best possible replication of the visual illusion*.” If model parameters are adjusted for each illusion, then a model can hardly claim to propose a plausible mechanism for explaining brightness perception. This is exactly the reason why so far no definite model has been found – each published mechanism/model explains only a subset of visual illusions (with constant held model parameters). And their failures are sometimes reveal where our understanding is yet incomplete.

The choice of an abstraction level for modeling depends on the considered mechanism (i.e., the computational hypothesis). This means that the parameter space (and the dimensionality of each model) should be as low as possible. Each parameter adds another degree of freedom which might not add to the overall understanding of the computational mechanism. For example, one could design a model at the neuronal level. But many of the published models ignore dendrites, and there is evidence that the dendrites are computationally more important than somas. Likewise, when considering stationary displays, modeling spike dynamics may add unnecessary

complexity. This means that no matter the abstraction level chosen for a model, there probably will be details at the neuronal level that are missed.

For our mechanism (decorrelation or response equalization, respectively), we believe that the description level is adequate.   Our processing is mainly local, where only ZCA samples image patches across the whole visual space. We did not make any claims about where ZCA might take place in the visual system. It may not be implemented in V1, though, but one cannot exclude for example, the retina or visual areas with lower resolution than V1. Local processing is certainly compatible with low-level visual processing at the neuronal level.

As with many similar models, the parameters for our model were found by trial-and-error in order to predict the maximum number of illusions. With corresponding computational resources, one would be able to systematically parse the parameter space, given an adequate measure of "success".

We improved the description of model parameters and their specific choice (where possible) in the methods section.

We also justified our model better in the introduction. Specifically, filling-in architectures (brightness perception models based on neuronal spreading of activity) are equally plausible as multi-scale filtering models, but more complicated at the mathematical level. Multi-scale models are largely based on the idea that the brain creates an identical representation (up to illusions) of the external world. Multi-scale models typically do not address that brightness perception involves the segregation of illumination and reflectance. Filling-in models, on the other hand, hold that neuronal spreading assigns homogeneous properties to surfaces in the visual space (e.h. lightness, depth, color, etc.).

More than that, you claim in the conclusion that you have principled derivation of your model based on predictive coding, but I do not see that clearly enough in the Methods section. I see two parts: one encoding the image into a compressed edge-like representation and regularized using some prior knowledge, then an iterative reconstruction (decoding?) inducing the brightness illusion. See for instance work from Spratling for similar architectures. This would be a great contribution of your paper.

From your comment and from those of the other reviewers, we recognized that the terms "*predictive coding*" and "*normalization*" are too general descriptions for our proposed mechanisms. In order to reduce the level of confusion by comparing our approach with others, and in order to avoid a certain degree of disappointment as a consequence of creating too high expectations associated with these terms, we finally decided to replace them with more detailed descriptions such as gain control, response equalization, or decorrelation. We hope that you agree with us that the latter terms are more adequate to describe our mechanisms.

I have some other points:
- embed your figures within the manuscript. It is tedious to go back and forth.

While we cannot agree more, this is the style demanded by PLoS.

- Figures appear at a low quality, especially figure 1. Use a vectorial format like PDF or EPS.

Thank you for the suggestion, we will do so.

- how do you justify physiologically and mathematically the existence of the two pathways (contrast + luminance) ?

The pathways are similar to the *Boundary Contour System* (BCS)/*Feature Contour System* (FCS) pathways that are proposed with most filling-in type models. We explained that better in the introduction and provided also some references which can be interpreted as supporting this hypothesis (please refer to the corresponding comment from reviewer 1 and our reply).

Mathematically, the filling-process proceeds faster (greater distance per unit time step) at a coarser scale, and the non-zero luminance response of the Contrast-Luminance channel further accelerates the convergence. We added this also to the methods section 2.3. Furthermore, the contrast-only channel reduces artifacts, what is illustrated with the new Figure 6, and also better described in section 2.3.

- you use flat, rectangular coordinates, not a retinotopic log-polar mapping. Specifically, you would not have an effect of scale as your Gabor filters have only one scale. For instance, the brightness illusion in the Hering grid (fig 16) is greater in the periphery - could you justify that?

Everything you said is of course correct. An important added-value-feature of our model is its capacity for real-world image processing, which is not easy at least with filling-in architectures. This means that it has a certain relationship with image processing algorithms, and therefore a log-polar mapping would not make much sense in this context (at least for static displays). Back to physiology, our model should be considered as describing information processing related to the fovea, where cones connect to ganglion cells in a 1:1 fashion, that is at the highest resolution level. Therefore, our model could not describe that the illusory gray spots which are perceived with the Hering/Hermann grid appear more intense in the periphery. Your comment also addresses the notion of viewing distance (or equivalently spatial resolution of the input images), which we did not address. To the best of our knowledge, the only published model that explicitly accounts for viewing distance is that of Otazu and coworkers. Furthermore, the classical explanation of the gray spots is based on lateral inhibition, what would explain the enhanced effect in the periphery (where receptive fields are bigger). However, lateral inhibition cannot explain why the spots disappear with a corrugated grid (Figure 18 in our revised manuscript) as well as other inconsistencies [1].

With respect to scale, we actually use two scales (0.25 and 0.125 cycles per image, respectively). Comment 4 of reviewer 1 raised a similar issue, so please see our corresponding reply. In short, bandpass filters like Gabor functions do not have a hard cut off with frequency, so they will respond also to other than their preferred spatial frequency, but with decreased amplitude. Of course, with a multi-scale decomposition, an image cannot be fully recovered with just two sets of filters with relatively narrow frequency bandwidth. However, with filling-in architectures, it is possible to recover images from high-pass information [2], and our model with its two scales is no exception (see Figure 22 in the revised manuscript).

[1] Schiller, P. H., & Carvey, C. E. (2005). The Hermann grid illusion revisited. Perception. https://doi.org/10.1068/p5447

[2] Keil, M. S., Cristóbal, G., Hansen, T., & Neumann, H. (2005). Recovering real-world images from single-scale boundaries with a novel filling-in architecture. *Neural Networks*. https://doi.org/10.1016/j.neunet.2005.04.003

- Energy map, S2 : from Parseval theorem, the local energy in Eq 10 is the sum of the spectral energy of each Gabor, and thus that from a DoG ?

Not exactly – because computation of local energy involves nonlinear operations (square and square root).

The idea can be illustrated by assuming a Laplacian kernel as DoG filter. So, in Matlab notation, *L=[0 1 0;1 -4 1; 0 1 0]*. The kernel with opposite polarity is *iL= [0 -1 0;-1 +4 -1; 0 -1 0]*. Therefore, convolution of L with an image patch *[0 a 0; q c r; 0 b 0]* gives $a+b+q+r-4c$, and convolution with iL gives the same result with opposite sign. Computing some kind of local energy for the two Laplacians therefore would give $\sqrt{(iL^2+L^2)}=\sqrt{2}\cdot|a+b+q+r-4c|$ (choosing the positive sign). Another possibility would be to compute $\sqrt{iL^2}+\sqrt{L^2}=2\cdot|a+b+q+r-4c|$. The result in both cases is proportional to $|a+b+q+r-4c|$.

Now we can define kernels corresponding to "*mini-Gabor*" functions (considering only horizontal/vertical orientations for simplicity). Assume the <u>even</u> kernel to be *[-1 2 -1] = - [1 -2 1]* (row vectors) and the <u>odd</u> oriented kernel as being *[0 -1 1]*. It is straightforward to show that adding two even kernels with perpendicular orientation gives L, and adding 4 odd kernels with different orientation (with -1 as center) gives also L (analog considerations for iL). Notice that the even kernel can be constructed from two odd kernels with one odd kernel having 180 degree orientation with respect to the other.

Now, for computing local energy with these kernel, we first convolve each of them with the image patch (index is orientation in degrees). For the odd kernels, the center is assumed to be fixed at -1

$$odd_0=r-c \quad , \quad odd_{90}=a-c \quad , \quad odd_{180}=q-c \quad , \quad odd_{270}=b-c$$

and for the even kernel *[-1 2 -1]* the convolution with the image patch gives

$$even_0=even_{180}=q+r-2c \quad , \quad even_{90}=even_{270}=a+b-2c$$

The local energy at the respective orientations therefore is

$$\mathrm{LE}_0=\sqrt{(r-c)^2+(q+r-2c)^2} \quad , \quad \mathrm{LE}_{90}=\sqrt{(a-c)^2+(a+b-2c)^2} \quad , \quad \mathrm{LE}_{180}=\sqrt{(q-c)^2+(q+r-2c)^2} \quad ,$$

$\mathrm{LE}_{270}=\sqrt{(b-c)^2+(a+b-2c)^2}$ (with identical results for the opposite polarities, that is the center of the odd kernel is fixed at +1, and the even kernel is *[-1 2 -1]*).

The final local energy is the sum of the oriented local energies (with a factor 2 due to polarities), and this is not equal to the result obtained with the Laplacian, which is proportional to $|a+b+q+r-4c|$

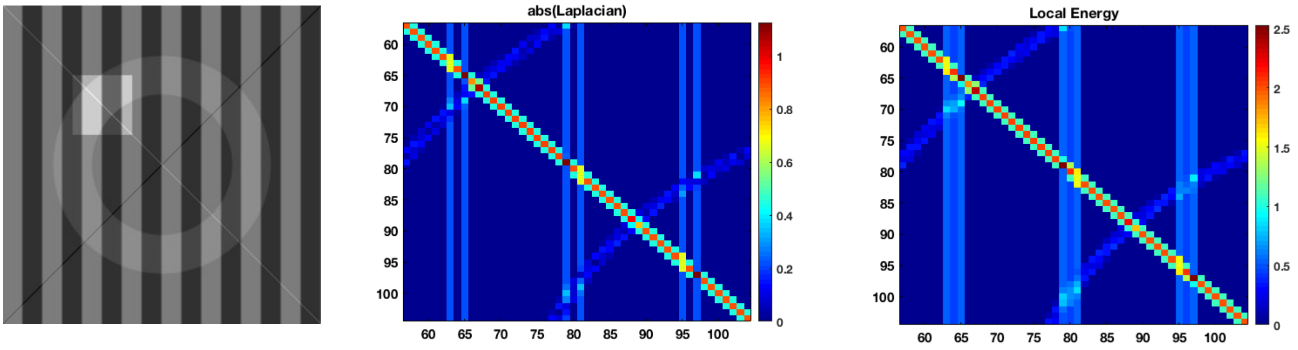

The figure shows a test image (courtesy of Hans du Buf), The first map shows $|a+b+q+r-4c|$ (absolute Laplacian) of the highlighted region of the input image. The second map is the local energy of the "*mini-Gabor*" kernels as described above. The responses to the diagonal line have the same pattern, while the responses to the vertical stripes and the disk boundary have different patterns. The local energy responses appear smoother than the responses of the Laplacian, while maintaining localization accuracy. Blurring of the Laplacian map would make the response patterns to disk and stripes more similar, but at the cost of spatial uncertainty.

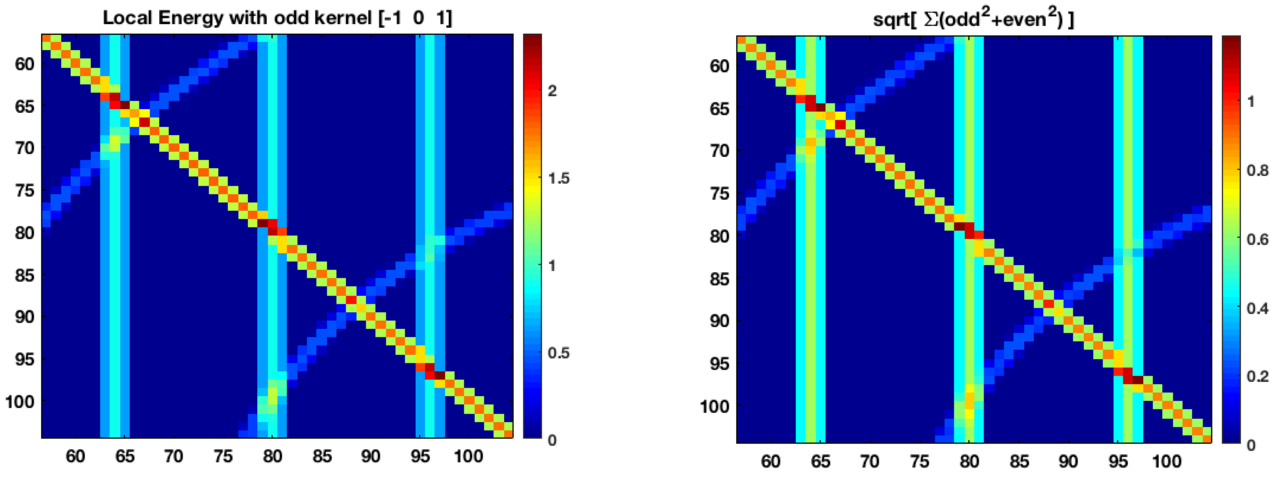

Finally, when using *[-1 0 1]* as the odd kernel, one obtains the local energy map on the left side. When taking the square root of the final sum (but not of the individual responses), one gets the local energy map on the right side (with the same odd kernel *[-1 0 1]*). The latter corresponds to the calculation of local energy in our model.

figure 12 context B: I would tend to see a convex object occluded by the square - could this explain the illusion?

(Note: Figure 12 has moved to Figure 14 in the revised version).

The classical explanations for this illusion is based on occlusion cue (we propose an alternative explanation with our model). It is usually assumed that the triggers for separating boundaries in depth are T-junctions, which are also effective in flat luminance displays. According to [1], the T-junctions or other occlusion cues are adequate to explain the direction of the brightness effect for context B, A, and C. However, each "theory" or computational model for explaining certain illusions has its limitations. For example, brightness assimilation seems to be independent of T-junctions (as with Shevell's Rings – Figure 1D in the revised manuscript), and does also occur when the occlusion cues are removed (as with Dungeon illusion – Figure 1C).

[1] *Güçlü, B., & Farell, B. (2005). Influence of target size and luminance on the White-Todorović effect. Vision Research.* https://doi.org/10.1016/j.visres.2004.10.025

-figure 20 : if you do a quantitative measurement, you should compare it with some other denoising model as a control. you do not need a 4 digit precision.

Thank you for the suggestion. We used a denoising method based on Tikhonov regularization as control [1], and noticed that our model removes noise worse than the control algorithm. However, noise removal is not the primary purpose of our results (albeit it is more a welcome by-product). We added corresponding information to Figure 22 and Section 3.4.

[1] Hearn, T.A., Reichel, L. Application of denoising methods to regularization of ill-posed problems. Numer Algor ***66,*** *761–777 (2014). https://doi.org/10.1007/s11075-013-9760-5*

L176: missing closing parenthesis

ok.

L209 : regarding the choice of Gabor filters, these contain a DC component - could you think of alternate filters that would better match to V1 cells?

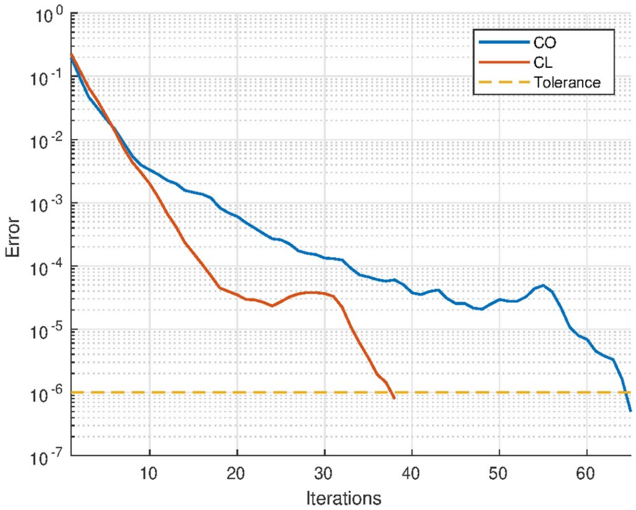

The DC component is very small (see new Figure 3). We used the unbalanced Contrast-Luminance (CL) kernels for two reasons. *First*, they take less iterations to convergence compared to the same filters with DC=0 (CO; figure on the left). *Second*, they are necessary for explaining *Chevreul's* illusion. The visual system somehow encodes relative luminance levels, for example in spike latencies [1]. Cells in the visual cortex also responds to temporal contrasts (i.e. changes of luminance in time) [2]. Although we do not consider response dynamics, we do not believe that a small non-zero DC response will rest too much credibility from our model. Or, the other way around, using a balanced Gabor function would probably not increase much its plausibility. Our model has a certain level of abstraction with respect to neuronal processing, and a putative small DC response might be implemented at the neuron level by different coneivable mechanisms.

[1] Rapid Neural Coding in the Retina with Relative Spike Latencies. Tim Gollisch and Markus Meister. Science 319(5866):1108-1111 (2008)

[2] Dai, J., & Wang, Y. (2012). Representation of surface luminance and contrast in primary visual cortex. *Cerebral Cortex*. https://doi.org/10.1093/cercor/bhr133

L224, l248, ... : LaTeX parenthesis `` or '' are mixed up. repeats throughout the text

ok (should be fixed during editing anyway).

L269 "sigmoidal function" > "sigmoid function"

ok.

L277 missing variable name in "The value of XXX acts "

ok

caption Fig4 "CL" > "LC"

ok – now Figure 5

caption Fig9 "Gain Control Maps" < "Gating Maps"

ok – now Figure 11

caption Fig19 "Garing" > "Grating"

ok – now Figure 21

Replies to Reviewer 3

This is a bold and interesting paper which presents a new model for the encoding of image brightness in perception, using a few broad, general computational principles aimed at reducing redundancy in boundary maps (p.20). The paper is bold because it attempts to account for a very wide range of brightness effects ('illusions') that have been described over a century or more of research. A long-standing challenge has been to account for 3 kinds of effects (contrast, assimilation, and filling-in) within a coherent framework. The model does a surprisingly good job of this, but the authors are also clear in describing where the model does not work, with some useful speculations in the Discussion about what else might be needed.

The paper covers a lot of ground, but is pretty compact in length. The Introduction is particularly useful in summarising both the (large) range of brightness effects studied in the literature, and the great variety of ideas that have been proposed to account for these effects.

The paper does well in presenting a verbal description of the model and its several stages, while reserving the mathematical details for the Appendix. Even so, I (not a computer scientist by trade) struggled to grasp the fillling-in process (Stage 3, 'brightness estimation'). In particular I could not see how, in the iterative process, the brightness image (z) got started, or what was being adjusted to reach a final result. Anything that would make this clearer to the reader would be valuable.

We like to thank you for this comment. Equation 5 is solved iteratively with the conjugate gradient method. The method starts with an image z_0 of random values. The weights of z_k are updated such that the value of the cost function $E(z_k)$ decreases with each iteration step. This gradient descent continues until a maximum of 100 iterations is reached, or until an error criterion is satisfied (Equation 6 in the original text). The filter responses R_g determine how the weights z_k are updated. Because R_g is higher at the edges, activity is iteratively propagated from the edges. Direction and orientation of activity spreading are determined by the odd-symmetrical Gabor filters that are used for encoding the image. Thus, image reconstruction (i.e., brightness estimation) proceeds according to a filling-in process.

We have added this explanation to Section 2.3. of the manuscript. Furthermore, we have moved Equation 6 (error criterion) to the appendix for an easier reading.

The paper is well-organized and generally clear, but English expression in the paper does not always flow as well as it might, so the paper would benefit from a 'light-touch' editing job by a native English speaker. I have made (below) some suggestions for syntax improvement, but more is needed.

Detailed commentary

References: Many references lack full information about volume & page numbers. Need checking.

Thank you for pointing this out. It was a *LaTex* compilation error. We fixed it.

L. 133, L. 371, L. 388, L. 768, L. 797: 'what' >>> 'that'. It would be best to check all instances of 'what'

Corrected

L 164: 'that' >>> 'which'

Corrected

L 224: as well as defining what 'g' is, state that Im(x,y) is the input luminance image (if this is so).

Updated

L. 264-271. Some reason and motivation for the various steps in this 'Gain Control Map' section would be helpful. And similarly for the subsequent section on "Gain control and normlization...". It would also useful to know where the chosen parameters (eg a, b, tau, filter parameters, alpha, threshold 'theta', etc) came from, and whether their values were critical.

Thank you for your suggestions. We progressively implemented some of the mechanisms and parameters of Equations 2, 3, and 4 by means of manual optimization. For instance, in Equation 2, we noticed that without thresholding, artifacts and noise tend to accumulate in the brightness prediction, but thresholding also serves to obtain a better sharpness after filtering. We have modified the corresponding part of the text and added more information for describing Equations 2, 3, and 4. Furthermore, we have added a new figure (Figure 5 in the revised manuscript) in the hope that it will lead to a better understanding of the gain control mechanism (formerly called normalization mechanism) of Equation 4.

L. 277: Something missing here: "The value of acts as an upper bound to the maximum activity"

Thank you, fixed.

L. 278-9: 'bigger (or smaller) than zero' >>> 'greater (or less) than zero'

Corrected.

Fig. 5 and later: black & blue are a poor choice of colours for the 'profile' curves in these figures; not very distinctive at all. My suggestion would be: black for input luminance, red for model brightness response. Plot the red first, then the black; that way the model's (red) deviations from luminance will appear very salient, as red segments.

Excellent suggestion, now the curves should be better discernible.

L. 326-7: you say "In this case, the major contribution to predicted brightness comes from the Gain Control Map (numerator of Eq 4)." But this seems very unclear to me, because the G(x,y) term appears in both the numerator & denominator; so some further explanation is required, as to why Eq 4 has the form it does, and what it achieves.

Thank you for pointing that out. We tried to explain that better by expanding and re-formulating the description of Equation 4; we added the new Figure 5; and we modified Section 2.4. (Scenarios).

Fig. 6: The input images (left column) clearly need a thin black outline to mark their full extent. At present, the upper input image apears to be a black square, rather than a white-black edge; and the lower image appears to have 3 regions, but actually has four.

We added gray picture frames to (now) Figure 8.

L. 366-368. On SBC, there appears to be a direct contradiction here: You say that "psychophysical studies report that the contrast effect is perceived less intense for smaller patches [60-64]", but Fig. 7D appears to show the weaker contrast effects at larger target sizes (not smaller), for both data & model. Something wrong here ? [You may perhaps be creating some confusion by switching between the use of "brightness" and "contrast" terms too often ?

Thank you very much for the observation. We corrected this. Of course, wider stimuli result in weaker simultaneous contrast, whereas narrower stimuli result in stronger effect.

And since the brightness values in Fig 7B can be both +ve & -ve, but those in panel D are purely positive, it's unclear what is being plotted in panel D: the 'brightness' of the right-hand patch, or the 'contrast' (ie difference ?) between left & right patches ? Or what ?]

Also, what is the published source for data in Fig. 7D ? Or are the 'data' actually model responses, not experimental data ?

The point symbols of (now) Figure 9D show the brightness difference in function of patch size as predicted by our model. The continuous line is a fitting function. We tried to make this clearer in the figure legend.

Fig 7, headings: Gating Map >>> Gain Control Map ?

Yes, corrected (now Figure 9).

Fig 10, axis labels: "Brigthness" >>> "Brightness"

Now Figure 12, we renamed to *Brightness Difference*.

L. 426: 'to a less detect' >>> 'to a lesser extent' ?

Corrected.

Fig 12, caption, last line: 'left square' >>> 'left disc' ?

Corrected.

L.657: "is dynamic filtering a global mechanism" >>> "dynamic filtering is a global mechanism"

Corrected

L. 663-671: Yes, a major strength is the range and variety of effects that can be reproduced by this model. And you also say that "for some of the illusions, we were able to replicate data from psychophysical experiments". To support this further, it would be good to see, for a few cases, actual comparisons between published experimental data and the model's predictions. Is that possible ? I realize that there might be a problem in expressing the model and data in the same metric, but perhaps there are ways around that ? Also, you mention that for all the cases examined here the same model parameters were used throughout. That is important, and could be mentioned much earlier in the paper.

Although it would we principally possible to overlay published data, and it would certainly add more credibility to the model if they could be reproduced exactly, it would require to write a corresponding software for sampling the data from the papers. To this end we would have required an additional one to two months of extension of the reviewing process because of software development. Considering this and the fact that we already had two deadline extensions (for various reasons), we think that this should be deferred to a future paper, and we hope that you agree with us. Therefore we prefer to relativize our original statement a little at this moment (i.e. formulating it more carefully): Instead of *"Additionally, for some of the illusions, we were able to* <u>*replicate data*</u> *from psychophysical experiments…"* we rephrased to *"Additionally, for some of the illusions, we were able to* <u>*reproduce the trend*</u> *for corresponding psychophysical data…"*

We were able to add the threshold contrast data for seeing the bright Mach band as measured by Ross and coworkers [1] to Figure 20C. These data were readily available, because one of us used them for the gradient system [2,3]

We furthermore added *"All of the following simulation results are based on the same set of model parameters. This means that model parameters were never changed."* to the beginning of the results section. We also added that model parameters were held constant at the end of the abstract.

[1] Ross, J., Concetta Morrone, M., & Burr, D. C. (1989). The conditions under which Mach bands are visible. *Vision Research*, *29*(6), 699–715. https://doi.org/10.1016/0042-6989(89)90033-3

[2] Keil, M. S. (2006). Smooth Gradient Representations as a Unifying Account of Chevreul's Illusion, Mach Bands, and a Variant of the Ehrenstein Disk. *Neural Computation*, *18*(4), 871–903. https://doi.org/10.1162/089976606775774705

[3] Keil, M. S., Cristóbal, G., & Neumann, H. (2006). Gradient representation and perception in the early visual system-A novel account of Mach band formation. *Vision Research*, *46*(17), 2659–2674. https://doi.org/10.1016/j.visres.2006.01.038

L. 679: define 'articulation pattern'

An articulation pattern can be created from a region with uniform luminance. The region is subdivided into small square patches. The luminance of each patch is randomly modulated according to a Gaussian random variable, with the mean value being the original luminance value, and the standard deviation being the modulation depth. The average luminance across the articulated pattern has to be identical to the luminance of the uniform region.

This explanation has been inserted into the discussion section as well.

Appendix, L. 740-744. Eqn 7 contains undefined terms x_hat, y_hat. And orientation 'theta' does not actually appear in the equation. Earlier (Eqn 2 & L.268) 'theta' appears as a threshold; this duplication would be best avoided by finding another symbol for the threshold.

We replaced the threshold symbol *theta* with *omega*.

Appendix, L. 748-752 are puzzling. First, gON and gOFF are not actually defined, though presumably they are the +ve and -ve regions of the Gabor RF, g, in Eqn 7.

We added a more detailed description to section 2.1.1 Contrast-only and Contrast-Luminance channels and Appendix S.1.

Second, and more importantly, the motivation for normalizing these 2 sub-fields separately is not described, and my guess is that it will have a different impact on the odd and even members of the Gabor pair. Does it ?

It depends. Assume two Gabor kernels with different symmetry and the same support. Also assume that the sum of the positive and negative kernel weights is positive, and this positive number is equal for both kernels. Then, both kernels will give an identical result when convolved with a homogeneous region. The whole thing looks different if the kernels are convolved with non-uniform regions that contain luminance patterns. But then, different results according to symmetry would be obtained anyway, no matter whether the kernels are unbalanced or not.

And because of this nonlinearity I think the pair of RFs are not actually a Gabor pair any more, and neither (strictly) is a Gabor function (ie. the product of a sinusoid and a Gaussian), unless alpha=0, and ||gON|| = ||gOFF||.

If the width of the Gaussian window (defined by its standard deviation) and the spatial frequency of the grating can vary independently from each other, varying the phase of the grating will often vary the DC value of a kernel. Nonzero DC responses of Gabor functions occur mostly with medium and high spatial frequency filters. However, if the standard deviation is suitably dependent on the spatial frequency, then the DC response will be near to zero independent of phase. So a Gabor function would still be a Gabor function if it had a non-zero DC response. A zero DC response is usually considered as a special property (of a set) of Gabor functions. For example, log-Gabor filters have a zero DC response by definition. In image processing, several methods for getting rid of the DC response were proposed, some of them including subfield manipulation as well. They are still referred to as Gabor filters.

Furthermore, in our case, the DC response of the filters in the Contrast-Luminance channel is very small. This mean that the contrast responses is dominant. We added Figure 3 to illustrate the luminance versus contrast response of the (non-)DC-free filters.

Summarizing, despite of our manipulation of the subfields, the filters still remain Gabor kernels - but with a specific property.

Third, the unbalanced combination of the on and off fields (when alpha>0) would make the RFs sensitive to luminance, as correctly implied by L.751. So presumably for the contrast-only channel, alpha=0, and not 0.1 as stated in L. 750?

This is correct. For the Contrast-Luminance kernels, $alpha = 0.1$, while we set $alpha=0$ for the Contrast-only filters.

With $\alpha = 0$, the area of both subregions (ON and OFF) of the filter is the same (ratio 1/1). This means that the DC component of the Gabor filter is zero (i.e., no sensitivity to mean luminance).

With $\alpha \neq 0$, the ratio between both areas becomes unbalanced (with ratio $1/(1-\alpha)$) and the Gabor filters are now sensitive to mean luminance.

Fourth, for the contrast-luminance channel, I imagine that you would want to build-in a greater response to dark than light (given what was said in the Introduction about asymmetry in the response to increments and decrements). But this would surely imply alpha<0 in Eqn 8, not alpha>0 (L 750) ? In short, a good deal of clarification is needed here.

Our first priority with the non-DC-free kernels was not to replicate physiological findings. Rather (in Equation 8) the portion of ON-region is kept constant, while α adjusts the OFF-region. With *alpha > 0* the contribution of ON-subfield is higher than that of the OFF-subfield. This means that the sign of luminance of a homogeneous version is preserved in the DC-response (we used luminance values from -0.5 to +0.5), because there are more positive (ON) values in the unbalanced kernel. This property is necessary for reconstruction, otherwise brightness would be predicted in the opposite direction. On the other hand, when convolved with luminance patterns, the ON amplitude (positive values) of the result will be higher than the OFF amplitude (negative values), and the difference corresponds to the DC response. The unbalanced kernels should only be considered as an abstract simple cell model. In the visual system, transmission of (relative) luminance levels could be accomplished by many possible mechanisms, for example via spike latencies [1]. Cells in the visual cortex also responds to temporal contrasts (i.e. changes of luminance in time) [2].

[1] Rapid Neural Coding in the Retina with Relative Spike Latencies. Tim Gollisch and Markus Meister. Science 319(5866):1108-1111 (2008)

[2] Dai, J., & Wang, Y. (2012). Representation of surface luminance and contrast in primary visual cortex. *Cerebral Cortex*. https://doi.org/10.1093/cercor/bhr133

Appendix, L. 755: more precisely, Rg,odd and Rg,even are not "a pair of Gabor filters" but "the responses from a pair of Gabor filters", aren't they ?

Yes this is true, thank you. We corrected that.
