# Supplementary material for: Dynamic decorrelation as a unifying principle for explaining a broad range of brightness phenomena
Source: PLoS Comput Biol. 2021 Apr 26;17(4):e1007907. doi: 10.1371/journal.pcbi.1007907 (PMC8102013; doi:10.1371/journal.pcbi.1007907)
Supplement: Attachment — Submitted filename: Replies_V3.pdf [file pcbi.1007907.s003.pdf]

Revision of “Dynamic Decorrelation as a unifying principle for explaining a broad range of brightness phenomena” by A. Lerer, H. Super, & M. S. Keil

We like to thank the reviewers and editor for their helpful comments.

Responses and list of changes:

>>> Editor #

Minor matters (indexed by line #):

197 “ifor” should be “for”

Corrected.

After all equations no indentation unless it’s a new paragraph

Corrected.

Fig 9 caption: typo: “continuous”

Corrected.

Fig 17 title: missing hyphen in “Craik-O’Brien-Cornsweet”

Corrected.

546 Suggestion: strike “We also wish to note that” before “We were unable…”

Corrected.

Fig 19 caption: No caps for “pyramid”

Corrected.

559 strike comma

Removed.

607 “gets” should be “becomes”

Improved.

700 “By all the cheers” sounds odd in English. Maybe “Despite all of these successes?’

Corrected as suggested.

782 “1 : 2” should be “1:2”

Corrected.

Reviewer's Responses to Questions

>>> Reviewer #1: The authors have Corrected. a very thorough and competent job in this revision. Happy to see it published.

We are glad to hear that, thank you very much! We wish also to thank you for your suggestions and comments.

>>> Reviewer #2: In this revision of their paper, authors propose changes in the form of writing but also in the scope of the results that are presented. As such, the manuscript is greatly improved from the first revision and requires only minor revisions before publication. I would like to congratulate the authors once again for this excellent work.

Thank you! And we also like to thank you for your suggestions for helping us to improve our manuscript.

First of all, the response to the various reviewers is detailed and precise. The decisions that have been taken, notably concerning the choice of title or the formalization of concepts in the paper (such as the use of gain normalization) have greatly contributed to improving the quality and clarity of the paper. Notably, both in the answer and in the manuscript, I appreciated the effort that was put into explaining with the model as many luminance illusions as possible while keeping the number of parameters in the model as low as possible.

Again, thank you very much.

One point that could be improved is the global explanation of the phenomena leading to luminance illusions throughout the different examples. For example for the Benary cross, the illusion is due to the redundancy of the contours and its modulation by the decorrelation mechanism. Insisting on the overall mechanism that produces the illusion will amplify the impact of the manuscript on its audience by explaining the mechanisms underlying the derivation of this particular model.

We think that the following short paragraph summarizes the principal mechanism of our model (and added it to the beginning of the conclusions):

One might ask whether the range of illusions that we successfully predict with our model can be attributed to a common mechanism. The answer is yes, and the underlying mechanism is dynamic filtering. Dynamic filtering acts to equalize the amplitude spectrum of a boundary map. In the spatial domain, thus, dynamic filtering depends on pattern redundancy (but not on activity). In this way, non-redundant patterns are enhanced (i.e., contrasted) compared to to redundant patterns (which are assimilated).

minor :
138 : "Barkan, [54], " > "Barkan [54] " l165 "White, [66], " > "White [66] " (see reviewers comment in previous revision)

Corrected.

223: g should be in math mode, not between apostrophes

Corrected.

225 : " image ." > " image."

Corrected.

774 : ", " at the begin of the line

Corrected.

>>> Reviewer #3: Overview

The authors have made extensive revisions to the MS that have improved its coherence and clarity. I think that their novel synthesis of spatial filtering, contrast gain control and image reconstruction ('filling-in') to account for brightness perception is plausible, interesting and important, and could have quite an impact on the field. I'm broadly satisfied with the paper as it now is, though a few corrections still need doing, as below. There may of course be others as well.

We also like to express our gratitude for your help in improving our manuscript.

Corrections:
Legend to Figs. 7A, 9C, 10C, 11C, 13A, 14A, 16A, 17A, 19C, 20B, 22B need correction (update) to the stated line colours: black, blue >>> red, black (arising from changes that I suggested previously for the figure plotting)

Corrected.

Fig 16A 'Duengeon' >>> 'Dungeon'

Corrected.
